# Prospective changes in sleep problems in response to the daily rest period among Japanese daytime workers: A longitudinal web survey

**DOI:** 10.1111/jsr.13449

**Published:** 2021-07-26

**Authors:** Hiroki Ikeda, Tomohide Kubo, Takeshi Sasaki, Yuki Nishimura, Xinxin Liu, Tomoaki Matsuo, Rina So, Shun Matsumoto, Masaya Takahashi

**Affiliations:** ^1^ National Institute of Occupational Safety and Health Japan Organization of Occupational Health and Safety Kawasaki Japan

**Keywords:** interval system, occupational health, quick return

## Abstract

The daily rest period (DRP) is the daily inter‐work interval and can include a sleep opportunity, leisure time, and other non‐work time. A longer DRP may allow workers to increase time in bed (TIB) and adjust sleep timing, and that may reduce sleep problems such as short sleep duration, sleep debt, social jetlag, and poor sleep quality. The present study examined the longitudinal association between the DRP and these sleep problems among Japanese daytime workers. The DRP, TIB on workdays, sleep quality (Pittsburgh Sleep Quality Index [PSQI]), sleep debt and social jetlag were measured in November 2016 (*n* = 10,000) and February 2019 (*n* = 3,098). Of these, 955 permanent daytime workers were divided into five groups based on the change in the DRP duration: shortened ≥2 hr, shortened ≥1 hr, no change (<1 hr), extended ≥1 hr and extended ≥2 hr. Linear mixed‐model analysis revealed significant interaction (group × time) effects on the TIB, PSQI score and sleep debt (all *p* < 0.001), but not on social jetlag (*p* = 0.476). Post hoc comparisons revealed that the TIB was decreased, and the sleep debt was increased in the shortened ≥2 hr group, whereas the TIB was increased and PSQI score was improved in the extended ≥2 hr group (all *p* < 0.01). These findings suggest that an extension of the DRP improves sleep quantity and quality but not sleep debt and social jetlag. Aside from extending the DRP, ensuring a sufficient sleep duration and adjusting sleep timing during the DRP may also be needed to prevent sleep problems.

## 1 INTRODUCTION

Sleep problems such as short sleep duration and poor sleep quality have negative effects on workers’ health. Short sleep duration and poor sleep quality are associated with an increased risk of type 2 diabetes (Cappuccio, D'Elia, Strazzullo, & Miller, 2010; Shan et al., 2015), cardiovascular disease (Itani, Jike, Watanabe, & Kaneita, 2017; Li et al., 2016; Sofi et al., 2014; Wang et al., 2016), and mental disorders (Baglioni et al., 2011; Zhai, Zhang, & Zhang, 2015). Furthermore, the joint association between short sleep duration and poor sleep quality are linked to workers’ sickness absence (Lallukka, Haaramo, Rahkonen, & Sivertsen, 2013) and disability retirement (Haaramo, Rahkonen, Lahelma, & Lallukka, 2012). The negative effects of sleep debt and social jetlag have also been reported. Sleep debt is defined as the cumulative hours of sleep loss with respect to subject‐specific daily need for sleep (Van Dongen, Rogers, & Dinges, 2003). It has been associated with workers’ sleepiness, depression, and presenteeism (Okajima, Komada, Ito, & Inoue, 2021). Meanwhile, social jetlag is the discrepancy between work (social time) and free (biological time) days (Wittmann, Dinich, Merrow, & Roenneberg, 2006). It has been associated with workers’ sickness absence (Lang et al., 2018) and poor work ability (Yong et al., 2016).

The European Union (EU) working time directive states that EU workers have the right to take ‘a minimum daily rest period (DRP) of 11 consecutive hours every 24 hr’ (European Parliament Council, 2003). In Japan, a ‘work interval system’, which requires employers to ensure workers get a certain interval of hours from the end of one workday to the start of the next (i.e. DRP; Ministry of Health, Labour, and Welfare, Japan, 2017), has been an obligation to make an effort to attain by the amendment of the ‘Act on Special Measures for Improvement of Working Hours Arrangements’, which came into effect on April 2019 (Ministry of Health, Labour, and Welfare, Japan, 2021). The DRP is expected to include activities and/or behaviours normally performed outside work hours, i.e. sleep, leisure time, commute time, and other non‐work time. Therefore, a longer DRP may result in a longer sleep opportunity. As a longer sleep opportunity could increase the time in bed (TIB) and allow a suitable adjustment of the sleep timing on workdays (social time) to non‐workdays (biological time), a longer DRP may improve sleep duration, sleep debt, and social jetlag. In addition, given that workers with many quick returns (short DRP of <11 hr) have frequent insomnia (e.g. Eldevik, Flo, Moen, Pallesen, & Bjorvatn, 2013), extending DRPs could also improve sleep quality. Our previous study examined the cross‐sectional association between DRP and these sleep problems and revealed that workers with shorter DRP had significantly shorter sleep duration, poorer sleep quality, and greater amounts of sleep debt and social jetlag (Ikeda et al., 2018, 2021). However, those previous studies were based on a cross‐sectional design, which does not address the causal links between DRP and sleep problems. A full understanding of the link between prospectively extending the DRP and the potential reduction in these sleep problems needs to be established.

The present study sought to address the research gap by examining the longitudinal association between DRP and sleep problems among Japanese daytime workers. We hypothesised that a longer DRP is longitudinally associated with fewer sleep problems.

## 2 METHODS

### 2.1 Survey and sampling

This study was approved by the Research Ethics Committee of the National Institute of Occupational Safety and Health, Japan (H2742 and H3027). The baseline survey was conducted in November 2016 and the follow‐up survey in February 2019. The surveys were conducted via a website by a third‐party research company (IDEA PROGGET Co., Ltd.) with a voluntary registrant database comprising almost 3.5 million individuals. In the baseline survey, the company randomly emailed invitations to participate in the survey to 60,000 registered workers. The individuals interested in the survey accessed the website Uniform Resource Locator (URL) enclosed in the email. There, they provided web‐based informed consent before they completed the survey. Subsequently, the participants received rewards points from the company. The first 10,000 workers (aged 20–64 years) who met the sample population criteria based on a composition ratio of sex, age group, and industry type as reported in the Labour Force Survey (Statistics Bureau, Ministry of Internal Affairs, and Communications, Japan, 2016) were recruited. In the follow‐up survey, participation requests were sent by email to the same 10,000 workers, and 3,098 of them completed the follow‐up survey.

**FIGURE 1:**
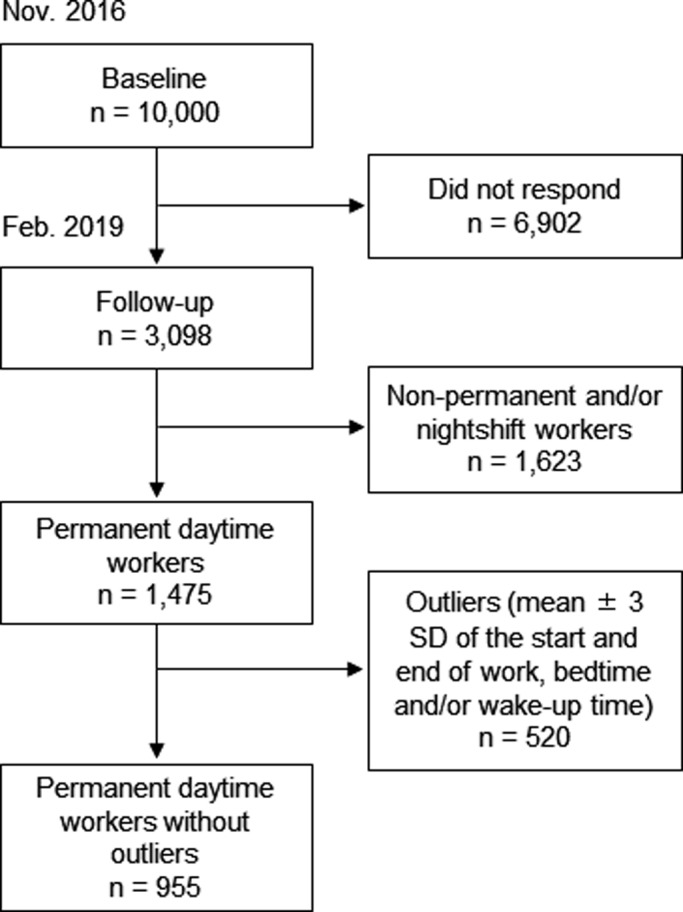
Participant enrolment. *SD*, standard deviation

As this study focussed only on permanent daytime workers, nightshift workers and workers with non‐permanent employment (e.g. part‐time or temporarily employed; *n* = 1,623) were excluded from subsequent analyses. In addition, permanent daytime workers with outlier data (±3 standard deviations [*SD*] from the mean of permanent daytime workers on the start of work, end of work, bedtime and/or wake‐up time; *n* = 520) were also excluded. The final sample size (i.e. permanent daytime workers without outliers) was 955 (Figure 1).

### 2.2 Data collection

Demographic data were collected, namely, sex, age (years), smoking habit (current smoker or ex‐ and non‐smoker), alcohol status (non‐consumption or more than once per week), employment type (permanent worker, part‐time worker, dispatched worker, contract employee, entrusted employee and other), presence or absence of night shift work (from 10:00 p.m. to 5:00 a.m.), and industry type (16 types).

The Workers’ Living Activity‐Time Questionnaire (Matsuo, Sasai, So, & Ohkawara, 2016), a self‐administered questionnaire, was used to ask about the averages in the previous month for the following: bedtime of the previous workday and non‐workday; wake‐up time on workdays and non‐workdays; presence or absence of the need to commute; if commuting, the start and end times of the commute; and the work beginning and end times. The DRP was calculated as the interval from ‘the end of working hours’ to ‘the beginning of working hours’. The TIB on workdays was calculated from ‘bedtime of the previous workday’ to ‘waking time on workday’. The TIB on non‐workdays was calculated from ‘bedtime of the previous non‐workday’ to ‘waking time on non‐workday’. Sleep debt was then derived by determining the difference in TIBs, which was calculated by subtracting the TIB on workdays from that on non‐workdays. The midpoint of sleep (mid‐sleep) was determined as the midway point between bedtime and wake‐up time (Sato‐Mito et al., 2011; Tavernier, Munroe, & Willoughby, 2015). The different mid‐sleep values, which were calculated by subtracting workday values from non‐workday values, were defined as social jetlag. In addition, the round‐trip commute time (the commute time for participants who did not commute was set to 0 hr) and leisure time (the time remaining after the TIB and round‐trip commute times were subtracted from the DRP) were calculated.

Sleep quality was measured using the Japanese version of the Pittsburgh Sleep Quality Index (PSQI‐J; Doi et al., 2000). The PSQI‐J presents 18 questions that evaluate seven component scores, namely, sleep quality, sleep onset latency, sleep duration, sleep efficiency, sleep disturbances, use of sleep medication, and daytime dysfunction in the previous month. The sum of the component scores is thus the PSQI‐J score (range: 0–21), which indicates sleep quality. Higher scores indicate more severe sleep complaints. The cut‐off point for primary insomnia was set at 5.5.

### 2.3 Analyses

Participants were divided into the following five groups according to their trajectory changes in the DRP duration: DRP shortened ≥2 hr (*n* = 27); DRP shortened ≥1 hr (≥1 DRP reduction <2 hr; *n* = 64); no change (DRP change <1 hr; *n* = 741); DRP extended ≥1 hr (≥1 DRP extension <2 hr; *n* = 83); and DRP extended ≥2 hr (*n* = 40).

To assess the longitudinal association between change in DRP and sleep problems, linear mixed‐model analysis with an unstructured covariance matrix was conducted for the TIB (workday and non‐workday), PSQI score, sleep debt, social jetlag, bedtime (workday and non‐workday), wake‐up time (workday and non‐workday), mid‐sleep (workday and non‐workday), leisure time, and round‐trip commute time. The groups (five groups) and survey times (baseline and follow‐up) were set as fixed effects and individual subjects as random effects. In addition, sex, age, smoking habit, and alcohol status were included in the model as fixed effects as confounding variables. The significance level was set at 0.05. Post hoc comparison for interaction effects was conducted by linear mixed‐model analysis with an unstructured covariance matrix in each group and adjusted by Bonferroni corrections (the significant level for the post hoc tests was set at 0.05/5 = 0.01). The random effect was individuals, and the confounding variables were sex, age, smoking habit, and alcohol status.

All statistical analyses were conducted using SPSS version 23.0 for Microsoft Windows (SPSS Software Inc.).

## 3 RESULTS

The demographic data are shown in Table 1. Compared with baseline, the female ratio was reduced >10% in the final analysis. The mean DRP at baseline and follow‐up in each group were 14.37 and 11.49 hr for the shortened ≥2 hr group, 14.25 and 12.93 hr for the shortened ≥1 hr group, 14.04 and 14.05 hr for no change group, 13.06 and 14.39 hr for extended ≥1 hr group, and 11.97 and 14.75 hr for extended ≥2 hr group (Table 2).

**TABLE 1:** Demographic data

	Baseline	Final analysis
	*N* = 10,000	*N* = 955
Sex (female), *n* (%)	4350 (43.5)	285 (29.8)
Age, years, *n* (%)		
20–29	1649 (16.5)	73 (7.6)
30–39	2331 (23.3)	231 (24.2)
40–49	2828 (28.3)	355 (37.2)
50–59	2302 (23.0)	255 (26.7)
60–64	890 (8.9)	41 (4.3)
Mean (*SD*)	42.9 (11.4)	44.2 (9.3)
Smoking habit, *n* (%)		
Ex‐ or non‐smoker	7426 (74.3)	695 (72.8)
Current smoker	2574 (25.7)	260 (27.2)
Alcohol status, *n* (%)		
Non‐consumption	5014 (50.1)	393 (41.2)
Once to more than six times per week	4986 (49.9)	562 (58.8)
Industry types, *n* (%)		
Agriculture and forestry	248 (2.5)	9 (0.9)
Construction	741 (7.4)	93 (9.7)
Manufacturing	1646 (16.5)	219 (22.9)
Information and communications	383 (3.8)	29 (3.0)
Transport and postal activities	553 (5.5)	41 (4.3)
Wholesale and retail trade	1623 (16.2)	184 (19.3)
Finance and insurance	267 (2.7)	39 (4.1)
Real estate and goods rental and leasing	197 (2.0)	34 (3.6)
Scientific research, professional, and technical services	347 (3.5)	34 (3.6)
Accommodations, eating, and drinking services	659 (6.6)	21 (2.2)
Living‐related and personal services and amusement services	355 (3.6)	25 (2.6)
Education, learning support	527 (5.3)	41 (4.3)
Medical, health care, and welfare	1322 (13.2)	95 (9.9)
Compound services	102 (1.0)	9 (0.9)
Services, not elsewhere classified	677 (6.8)	45 (4.7)
Government, except elsewhere classified	353 (3.5)	37 (3.9)

**TABLE 2:** Information of daily rest periods and sleep‐related variables on baseline and follow‐up in each group (n = 955)

Group	Change in DRP duration										Interaction, *p*
	Shortened ≥2 hr (*n* = 27)		Shortened ≥1 hr (*n* = 64)		No change (*n* = 741)		Extended ≥1 hr (*n* = 83)		Extended ≥2 hr (*n* = 40)		
Time	Baseline	Follow‐up	Baseline	Follow‐up	Baseline	Follow‐up	Baseline	Follow‐up	Baseline	Follow‐up	
Information of DRP											
DRP, hr, mean (*SD*)	14.37 (1.83)	11.49 (2.02)	14.25 (1.43)	12.93 (1.43)	14.04 (1.11)	14.05 (1.13)	13.06 (1.54)	14.39 (1.49)	11.97 (1.58)	14.75 (2.18)	‐
Prevalence of quick return (<11 hr), *n* (%)	0 (0)	8 (30)	0 (0)	5 (8)	12 (2)	15 (2)	5 (6)	1 (1)	11 (28)	0 (0)	‐
Sleep‐related variables, estimated marginal means (standard error of the mean)											
Time in bed on non‐workday	7.34 (0.20)	7.55 (0.26)	7.48 (0.17)	7.47 (0.17)	7.55 (0.05)	7.63 (0.05)	7.37 (0.15)	7.47 (0.15)	7.49 (0.22)	7.84 (0.21)	0.607
Round‐trip commute time	1.47 (0.18)	1.59 (0.21)	1.34 (0.12)	1.50 (0.14)	1.39 (0.03)	1.44 (0.04)	1.32 (0.10)	1.34 (0.12)	1.45 (0.15)	1.59 (0.17)	0.793
Leisure time on workday	6.39 (0.31)	4.31 (0.34)	6.84 (0.20)	5.52 (0.22)	6.26 (0.06)	6.09 (0.07)	5.49 (0.18)	6.65 (0.19)	4.79 (0.26)	6.85 (0.28)	<0.001
Bedtime on the previous workday	23.78 (0.24)	24.14 (0.25)	23.86 (0.16)	24.08 (0.16)	23.84 (0.05)	23.81 (0.05)	23.88 (0.14)	23.77 (0.14)	24.28 (0.20)	23.89 (0.20)	0.002
Wake‐up time on workday	6.29 (0.19)	5.71 (0.18)	6.03 (0.13)	6.06 (0.12)	6.22 (0.04)	6.29 (0.04)	6.23 (0.11)	6.23 (0.11)	6.09 (0.16)	6.25 (0.15)	<0.001
Bedtime on the previous non‐workday	24.10 (0.26)	24.07 (0.26)	24.04 (0.17)	24.05 (0.17)	24.14 (0.05)	24.05 (0.05)	24.10 (0.15)	23.86 (0.15)	24.56 (0.22)	24.19 (0.22)	0.235
Wake‐up time on non‐workday	7.43 (0.29)	7.62 (0.29)	7.51 (0.19)	7.52 (0.19)	7.70 (0.06)	7.68 (0.06)	7.47 (0.17)	7.33 (0.17)	8.05 (0.24)	8.04 (0.24)	0.654
Mid‐sleep on workday	27.03 (0.18)	26.92 (0.19)	26.94 (0.12)	27.07 (0.12)	27.03 (0.03)	27.05 (0.04)	27.06 (0.10)	26.99 (0.11)	27.18 (0.15)	27.07 (0.15)	0.190
Mid‐sleep on non‐workday	27.77 (0.24)	27.85 (0.25)	27.77 (0.16)	27.78 (0.16)	27.92 (0.05)	27.86 (0.05)	27.78 (0.14)	27.60 (0.14)	28.30 (0.20)	28.11 (0.20)	0.317

DRP, daily rest period.

Covariates included sex, age, smoking habits, and alcohol status.

**FIGURE 2:**
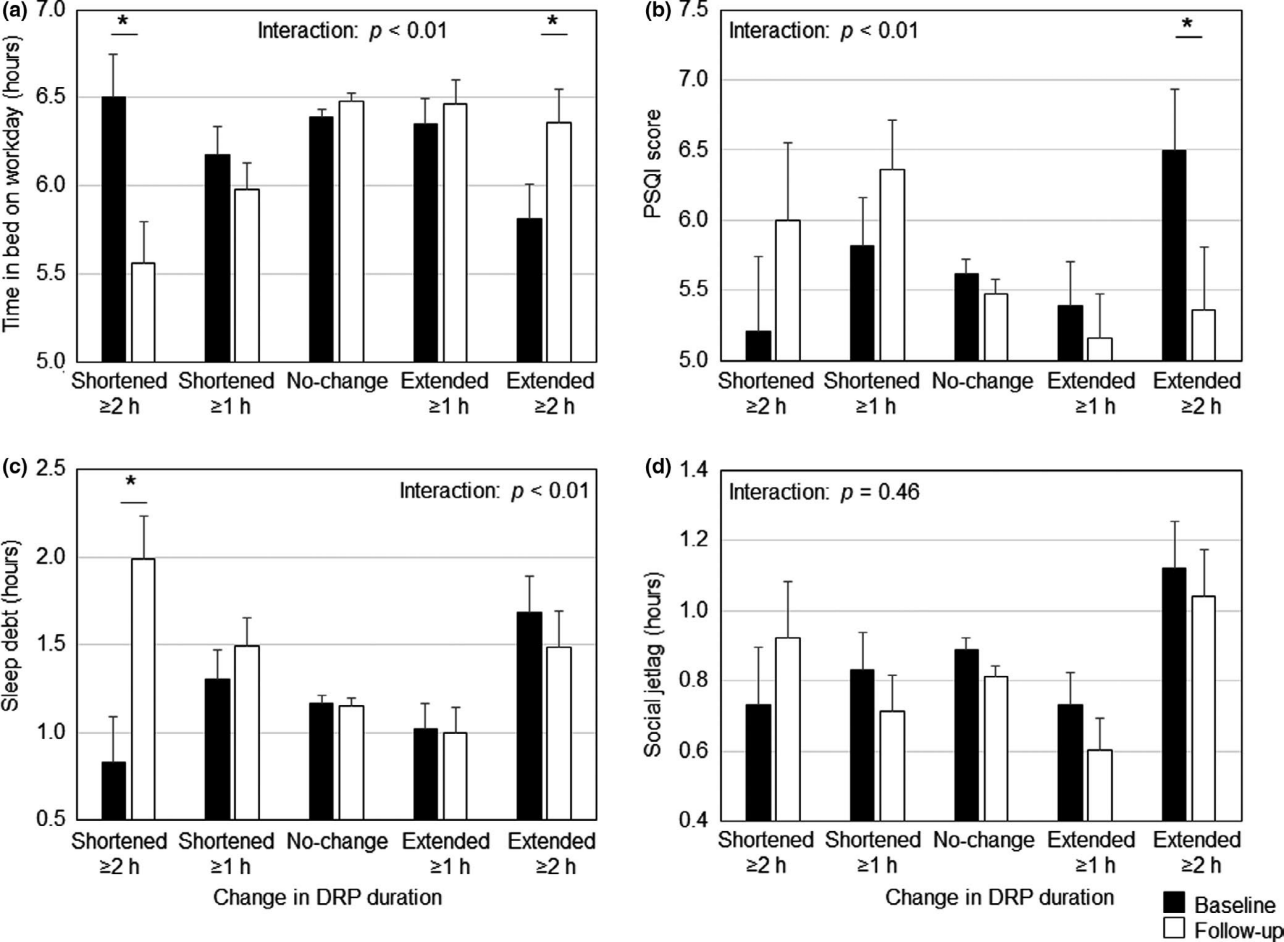
Estimated marginal means (standard error of the mean) of time in bed on workdays, Pittsburgh Sleep Quality Index (PSQI) score, sleep debt and social jetlag of baseline and follow‐up data in each group (*n* = 955). Covariates were sex, age, smoking habits, and alcohol status. DRP, daily rest period. *p < 0.01

Figure 2 shows the changes in (a) the TIB on workdays, (b) PSQI score, (c) sleep debt, and (d) social jetlag in each group. The linear mixed‐model analysis for the TIB on workdays revealed significant interaction effects (*F*[4, 949] = 8.356; *p* < 0.001), whereas post hoc comparisons showed that the TIB on workdays was significantly decreased in the DRP shortened ≥2 hr group (*F*[1, 27] = 12.757; *p* = 0.001) and increased in the DRP extended ≥2 hr group (*F*[1, 44] = 8.555; *p* = 0.005). The linear mixed‐model analysis for the PSQI score indicated significant interaction effects (*F*[4, 940] = 4.770; *p* = 0.001), while post hoc comparisons revealed that the PSQI score was significantly improved in the DRP extended ≥2 hr group (*F*[1, 53] = 11.316; *p* = 0.001). The linear mixed‐model analysis for the sleep debt signified significant interaction effects (*F*[4, 950] = 5.592; *p* < 0.001), whereas post hoc comparisons demonstrated that the sleep debt was significantly increased in the DRP shortened ≥2 hr group (*F*[1, 28] = 11.780; *p* = 0.002). However, the linear mixed‐model analysis for the social jetlag revealed non‐significant interaction effects (*F*[4, 950] = 0.896; *p* = 0.466).

Table 2 shows the changes in the TIB on non‐workdays, round‐trip commute time, leisure time, bedtime of previous workday and non‐workday, wake‐up time on the workday and non‐workday, and mid‐sleep on workday and non‐workday in each group. For leisure time, the linear mixed‐model analysis revealed significant interaction effects (*F*[4, 949] = 66.706; *p* < 0.001), whereas post hoc comparisons showed it was significantly decreased in the DRP shortened ≥2 hr (*F*[1, 29] = 31.946; *p* < 0.001), shortened ≥1 hr (*F*[1, 75] = 34.922; *p* < 0.001), and no change (*F*[1, 846] = 9.427; *p* = 0.002) groups. However, post hoc comparisons showed the leisure time was increased in the DRP extended ≥1 hr (*F*[1, 101] = 78.139; *p* < 0.001) and ≥2 hr groups (*F*[1, 41] = 32.153; *p* < 0.001). The linear mixed‐model analysis for the bedtime of the previous workday revealed significant interaction effects (*F*[4, 950] = 4.259; *p* = 0.002), whereas post hoc comparisons revealed that the bedtime of the previous workday became significantly later in the DRP shortened ≥2 hr group (*F*[1, 35] = 7.622; *p* = 0.009). Although the linear mixed‐model analysis for the wake‐up time on workdays revealed a significant interaction effect (*F*[4, 944] = 5.060; *p* < 0.001), post hoc comparisons revealed no significant differences between baseline and follow‐up among all groups (all *p* > 0.01). Non‐significant interaction effects (*p* > 0.05) were found for the TIB on non‐workdays, round‐trip commute time, bedtime of the previous non‐workday, wake‐up time on non‐workday, mid‐sleep on workday, and mid‐sleep on non‐workday.

In addition, a significant difference was seen between baseline and follow‐up in the leisure time in the no change group. Therefore, we divided the participants in the no change group into three groups according to the change in DRP duration (DRP shortened <1 hr group [*n* = 298]; completely same group [*n* = 126]; and DRP extended <1 hr group [*n* = 317]) and conducted linear mixed‐model analysis (3 groups × 2 times). The results revealed significant interaction effects (*F*[2, 736] = 19.880; *p* < 0.001). Post hoc comparisons revealed significant differences in the DRP shortened <1 hr group (estimated marginal means [standard error of the mean]: baseline = 6.32 [0.09]; follow‐up = 5.80 [0.10]; *F*[1, 333] = 25.729; *p* < 0.001), but not in the completely the same group (baseline = 6.37 [0.14]; follow‐up = 6.34 [0.15]; *F*[1, 157] = 0.299; *p* = 0.586), and DRP extended <1 hr group (baseline = 6.21 [0.09]; follow‐up = 6.34 [0.09]; *F*[1, 370] = 3.197; *p* = 0.075).

## 4 DISCUSSION

The present study examined the longitudinal association between DRP and sleep problems among Japanese daytime workers, and our findings thus partially support our hypothesis that a longer DRP is longitudinally associated with fewer sleep problems.

The DRP includes sleep opportunity, and a longer sleep opportunity may allow for a longer TIB. The previous cross‐sectional studies found that a longer DRP was associated with a longer TIB (Ikeda et al., 2018) and sleep duration (Kubo et al., 2018) on workdays. Therefore, a change in DRP may influence the actual TIB. The present longitudinal study showed that a change in DRP influenced the TIB on workdays, that is, a longer DRP increased the TIB on workdays. However, the TIB on workdays was significantly changed in the DRP extended/shortened ≥2 hr groups, but not in the DRP extended/shortened <2 hr groups. In contrast, leisure time was significantly changed in the DRP extended/shortened ≥2 and ≥1 hr groups. A previous cross‐sectional study found that the DRP correlated with the TIB and leisure time on workdays; however, the correlation between the DRP and the TIB was significantly weaker compared with leisure time (Ikeda et al., 2018). These results suggest that when the DRP change is small, the change may be mainly compensated by leisure time. However, if the change is large, the change may be reflected in both the leisure time and TIB. Previous studies reported that a short sleep duration has negative effects on workers’ health. The National Sleep Foundation (Hirshkowitz et al., 2015) recommended 7–9 hr and not <6 hr of sleep for adults (aged 26–64 years). Therefore, if workers have <6 hr of sleep, the workers may need to prioritise getting the recommended sleep duration over getting leisure time during DRP.

Our previous cross‐sectional study found that the DRP was associated with sleep debt, i.e. workers with a shorter DRP reported significantly greater amounts of sleep debt than workers with a longer DRP (Ikeda et al., 2021). However, the present longitudinal study showed that although a shortened DRP increased sleep debt, the extension of the DRP did not reduce sleep debt. In the present study, sleep debt was calculated by subtracting the TIB on workdays from the TIB on non‐workdays. Habitual short TIB on workdays may increase the TIB on non‐workdays (sleep rebound). Therefore, the difference between the TIB on workdays and non‐workdays (i.e. sleep debt) in the DRP shortened ≥2 hr group may have been significantly increased in follow‐up compared with baseline. On the other hand, the extension of DRP may advance the bedtime of the previous non‐workday, which may increase the TIB on non‐workdays. Therefore, in the DRP extended ≥2 hr group, an increase of the TIB on workdays and non‐workdays occurred in the follow‐up, and the differences (sleep debt) may not have been significantly reduced at follow‐up compared with those at baseline.

The increase of sleep opportunity with the extension of the DRP may help adjust the sleep timing and decrease social jetlag. Our previous cross‐sectional study found a relationship between the DRP and social jetlag, i.e. workers with a shorter DRP reported significantly greater amounts of social jetlag than workers with a longer DRP (Ikeda et al., 2021). However, in the present study, no longitudinal relationship was found between the DRP and social jetlag, as well as with mid‐sleep on workdays. One possible cause is that the workers may not have changed their sleep phases even if their DRP (sleep opportunity) was extended or shortened. Therefore, to reduce the social jetlag, the workers may need to adjust their workday sleep timing during an extended DRP to that of their non‐workday sleep schedule, i.e. reducing the discrepancy in sleep timing between social time and biological time. Other possible causes include offsets in the sleep phase changes, as sleep phase changes may differ on the chronotypes. For example, the sleep phase may advance in the morning type but delay in the evening type (Roenneberg et al., 2007). Further studies are needed to solve the issue.

Previous studies found that DRP and quick returns are associated with sleep quality (Eldevik et al., 2013; Ikeda et al., 2018). In the present study, the analysis of the longitudinal association between the DRP and sleep quality showed that the workers who extended their DRP showed improved sleep quality. However, the cause could not be determined in the present survey. One possible reason is the reduction of stress by extended leisure time and/or shortened working hours on workdays rather than the extension of the DRP per se. Further studies would be needed to clarify the causes.

The present study has several limitations. First, sleep problems were determined based on self‐reported outcomes. Second, the data were collected using a web survey, thus, sampling bias may have occurred. Third, the present study did not collect data on how workers used their leisure time. Leisure time may include unpaid work such as housekeeping and caregiving, or sometimes working overtime at home during the DRP, the influence of which on sleep problems will not have been assessed (Arlinghaus & Nachreiner, 2014; Byun, Lerdal, Gay, & Lee, 2016). Fourth, we did not collect data on some confounders related to sleep problems such as body mass index, consumption of caffeine, and current marital or live‐in partner status. Finally, in the present study, the DRP of 22.4% of workers changed (≥1 hr) after 2 years. Some possible reasons for this have been identified. For one, the Labour Force Survey (Statistics Bureau, Ministry of Internal Affairs, and Communications, Japan, 2021) reported that >3 million workers changed jobs each year from 2016 to 2019. In addition, although both baseline and follow‐up surveys were conducted before the work interval system came into effect, the number of companies with newly introduced work interval systems had slightly increased: 1.4% (*n* = 4,432), 1.8% (*n* = 3,697), 3.7% (*n* = 4,127) and 4.2% (*n* = 4,191) in 2017, 2018, 2019 and 2020, respectively (Ministry of Health, Labour, and Welfare, Japan, 2020). The job changes or this policy implementation in the companies are among the possible causes of the DRP changes. However, some conditions would have served to change the DRP, e.g. the workers were unable to work as hard as they used to after an illness or accident. In these cases, sleep problems are unlikely to be improved even if the DRP were extended. Further interventional studies are needed to clarify the issue.

Despite these limitations, the present study established a longitudinal association between the DRP, which contains sleep opportunity, and sleep problems in Japanese daytime workers. Although the work interval system has been promoted in Japan, only 4.2% of 4,191 Japanese companies have adopted the system (Ministry of Health, Labour, and Welfare, Japan, 2020). Introduction of the work interval system and extension of the DRP may help improve sleep quantity and quality. In addition, ensuring the recommended sleep duration and adjusting sleep timing during the DRP may reduce sleep debt and social jetlag. Overall, these measures may reduce workers’ sleep problems and related health issues.

## CONFLICT OF INTEREST

The authors have no conflicts of interest to declare.

## AUTHOR CONTRIBUTION

HI designed the study. TK, TS, XL and TM conducted the survey. HI and YN carried out analyses. HI wrote the main text. MT supervised the study and managed the research grant. All authors contributed to the final version of the manuscript.

## Data Availability

Research data are not shared because we did not inform the participants of the data transparency nor declare the possibility on the institutional review board.
